# Hypolipidemic Effects and Preliminary Mechanism of Chrysanthemum Flavonoids, Its Main Components Luteolin and Luteoloside in Hyperlipidemia Rats

**DOI:** 10.3390/antiox10081309

**Published:** 2021-08-20

**Authors:** Jihan Sun, Zhaodan Wang, Lin Chen, Guiju Sun

**Affiliations:** 1 Key Laboratory of Environmental Medicine and Engineering of Ministry of Education, and Department of Nutrition and Food Hygiene, School of Public Health, Southeast University, Nanjing 210009, China; sjhunian@163.com (J.S.); wdeairen07@163.com (Z.W.); friendlin@126.com (L.C.); 2 Technology Research Center of Characteristic Biological Resources in Northeast of Chongqing, College of Biology and Food Engineering, Chongqing Three Gorges University, Chongqing 404130, China

**Keywords:** chrysanthemum flavonoids, luteolin, luteoloside, hyperlipidemia, hepatic steatosis

## Abstract

This study aimed to investigate the key constituents and preliminary mechanism for the hypolipidemic activity of chrysanthemum flavonoids. Hyperlipidemia (HPL) rats were divided into five groups: the model control group (MC); Chrysanthemum flavone intervention group (CF); luteolin intervention group; luteoloside intervention group and simvastatin intervention group. The body weight, organ coefficient, serum lipids, antioxidant activity, and lipid metabolism enzymes were detected. Hematoxylin and eosin (H&E) staining was used to observe the liver and adipose tissue. Chrysanthemum flavonoids, luteolin, and luteoloside can reduce the weight and levels of total cholesterol (TC), triglycerides (TG), and LDL-C, and increase the level of HDL-C in the blood and reduce liver steatosis. Indicators of liver function (AST, ALT, and ALP) improved. The antioxidant activity (GSH-Px, CAT, SOD) and enzymes associated with lipid catabolism (FAβO, CYP7A1, and HL) increased, while lipid peroxidation products (MDA) and enzymes associated with lipid synthesis (FAS, HMG-CoA, and DGAT) decreased. Chrysanthemum flavonoids had a better effect on the antioxidant level and lipid metabolism-related enzyme activity. There was no significant difference in the effects of the chrysanthemum flavonoids, luteolin, and Luteoloside on improving blood lipids and hepatic steatosis—mechanisms that may be related to antioxidant levels and regulating enzymes involved in the metabolism of fatty acids, cholesterol, and triglycerides in the liver. However, chrysanthemum flavonoids had a stronger antioxidant and lipid metabolism regulation ability, and the long-term effects may be better.

## 1. Introduction

Flavonoids, anthocyanins, alkaloids, phenolic acids, and other phytochemicals in flowers have anti-microbial, antioxidant, anti-inflammatory, anticancer, anti-obesity, and neuroprotective effects, which provide the theoretical basis for their health benefits [1]. In the latest Chinese pharmacopeia (2015 edition), among 521 Chinese herbal medicines, 28 were flower-derived [2]. Chrysanthemum (*Chrysanthemum morifolium Ramat.*), the most representative flower-derived dietary herbal medicine, has been used for more than 2000 years in China and has been recorded in the Chinese pharmacopeia since 1967 [3]. Its flower head, named Juhua in Chinese, is widely used in traditional Chinese medicine, as a food supplement, and in herb tea [4].

Flavonoids are the major compounds in chrysanthemum and are responsible for their colorful appearance. Most of them are luteolin, apigenin, acacetin, diosmetin, or their glycosidic derivatives [5,6]. Many analytical technologies have been applied for quality control of Chrysanthemum. TLC and HPLC/UPLC are essential methods for quality control of herb medicines or food supplements. Six compounds, including luteolin, chlorogenic acid, 3,5-di-O-caffeoylquinic acid, luteolin 7-*o*-β-d-glucopyranoside, kaempferol, and acacetin were identified by reverse-phase thin layer chromatography (RP-TLC) [7], and the contents of chlorogenic acid, 3,5-di-o-caffeoylquinic acid, and luteolin 7-*o*-β-d-glucopyranoside are no less than 0.20%, 0.70%, and 0.08%, using HPLC combined with a UV detector for determination [8]. However, the weakness of TLC and HPLC/UPLC lies in their relatively low sensitivity and suitability for compounds with large quantities; LC-MS and GC-MS were applied for the analysis of minor compounds. Our previous studies have shown that chrysanthemum has a very high content of total flavonoids and total steroids in 12 edible flowers [9], and showed good antioxidant effects in vivo and in vitro [10]. Chrysanthemum is a popular traditional Chinese medicine used for cardiovascular diseases, especially hyperlipidemia and hypertension. Accordingly, the hypolipidemic and antihypertensive activities of its extracts by different solvents are active in varying degrees [11,12,13]. The hypolipidemic effect of chrysanthemum total flavonoids on hyperlipidemia in rats has been proven [14]. However, the pure compounds were not investigated to explain which constituents were the key attributes to the clinical therapy of hyperlipidemia. We determined the total flavonoids in chrysanthemum and found that the contents of luteolin and luteoloside in different varieties of chrysanthemum were very high, and there was a certain correlation between the antioxidant activity and the content of total flavonoids [15]. The flavonoids of chrysanthemum were isolated and purified by AB-8 macroporous adsorption resin and identified by LC-MS/MS. The flavonoids contained luteolin-7-glucoside, luteolin, apigenin, and apigenin-7-glucoside [16]. We also optimized the extraction method of chrysanthemum flavonoids [17] and established a high-performance liquid chromatography (HPLC) method for the determination of luteolin-7-O-glucoside in chrysanthemum [18].

In this study, we hypothesized that luteolin and luteoloside are the main components of chrysanthemum flavonoids that play a role in lowering blood lipids, and their mechanism of action is related to antioxidant activity. We used hyperlipidemia rats to investigate the lipid metabolism improving effects of chrysanthemum flavonoids and its main components, luteolin, and luteoloside, by focusing on the serum lipids, liver pathology, antioxidant activity, and enzymes related to lipid metabolism. The findings will help understand the key constituents and preliminary mechanisms for the hypolipidemic activity of chrysanthemum flavonoids.

## 2. Materials and Methods

### 2.1. Materials

The pure products of chrysanthemum flavonoids, luteolin, luteoloside, and simvastatin were all purchased from the Nanjing Zelang Pharmaceutical Company. Sodium carboxymethyl cellulose (CMC) solution (0.5%) was used to prepare gastric gavage fluid, and the concentrations of the chrysanthemum flavonoids, luteolin, luteoloside and simvastatin were 10 mg/mL, 5 mg/mL, 2.5 mg/mL, and 1 mg/mL, respectively, which was equivalent to the intervention dose chrysanthemum flavonoids of 100 mg/kg, luteolin 50 mg/kg, luteoloside 25 mg/kg, and simvastatin 10 mg/kg.

### 2.2. Hyperlipidemia Rats Model and Experimental Design

Sixty SPF male Sprague-Dawley rats (200 ± 9 g) were purchased from Nanjing Medical University, and the license was SYXK (SU) 2016-0014. The rats were housed five per cage in a room with a 12 h diurnal cycle and an ambient temperature of 22~24 °C. The animal ethics approval was 2015ZDSYLL004.0.

All animals were fed adaptively for one week by a D12450B diet and then randomly divided into a normal control group (NC, n = 10) fed by a D12450B diet, and a model group (n = 50) fed by a D12492 high-fat diet. The diet ingredients are shown in Table 1. After 4 weeks of modeling, the serum TG and TC in the model group were significantly higher than those in the normal control group (*p* < 0.05), and the modeling was judged to be successful.

According to the serum TG and TC, fifty male hyperlipidemia rats were randomly divided into five groups, including the model control group (MC); chrysanthemum flavone intervention group (CF); luteolin intervention group; luteoloside intervention group, and simvastatin intervention group. The different intervention solutions (diluted with 0.5 % CMC solution) were intragastrically administered to four intervention groups every day for 6 weeks at 10 mL/kg, and 0.5 % CMC solution was given to the normal control group and the model control group. Intervention doses for each group are shown in Table 2. The body weight was weighed every Monday morning.

### 2.3. Collection of Blood and Tissue Samples

At the end of the intervention, all rats were anesthetized with pentobarbital sodium and cervical decapitated after fasting for 12 h. Blood was collected from the femoral artery and centrifuged at 3000 rpm for 15 min. The serum was stored at −20 °C. Liver and total visceral fat (peri-testicular, peri-renal, mesenteric, and retroperitoneal fat) were rapidly separated and weighed. Feces and liver were frozen in liquid nitrogen and stored at −80 °C.

### 2.4. Biochemical Assays

TC, TG, high-density lipoprotein cholesterol (HDL-C), low-density lipoprotein cholesterol (LDL-C), apolipoprotein-A (Apo-A), apolipoprotein-B (Apo-B), aspartate aminotransferase (AST), alkaline phosphatase (ALP), and alanine aminotransferase (ALT) were determined by an automatic biochemical analyzer (Bechman coulter, America). The liver tissue was thoroughly mixed with normal saline at 1:9 (mg: μL), centrifuged at 3000 rpm for 10 min and the supernatant 10% liver homogenate was prepared. Glutathione peroxidase (GSH-Px), malondialdehyde (MDA), superoxide dismutase (SOD), and catalase (CAT) were respectively measured by colorimetry, TBA, hydroxylamine, and visible spectrophotometry methods. Liver homogenate (1 mL = 0.1 g) was prepared by mixing 10 mg of tissue with 100 microliters of PBS and then fatty acid synthase (FAS), cholesterol 7-alpha-hydroxylase (CYP7A), diacylglycerol acyltransferase (DGAT), fatty acid β oxidase (FAβO), 3-hydroxy-3-methylglutaryl-coenzyme A (HMG-CoA), and hepatic lipase (HL) were analyzed by commercial ELISA assay kits (Nanjing Jiancheng Bioengineering Institute, China) using an ELISA analyzer (RT-6100, Rayto, China).

Calculation of other evaluation parameters: The ratio of body fat= total fat weight(g)/body weight(g) × 100%; liver index = wet liver weight(g)/body weight(g) × 100%.

### 2.5. Pathological Observation of Liver and Peri-Testicular Fat

The liver and peri-testicular fat were fixed with 10% neutral formaldehyde for 48 h. Paraffin sections were prepared and stained with hematoxylin and eosin (H&E). The degree of hepatic steatosis was quantitatively analyzed by Image-pro Plus 6.0 (0 point was normal, 1 point was less than 5%, 2 points was 5–30%, 3 points was 30–50%, 4 points was 50–75%, and 5 points was greater than 75%). The number of adipocytes per unit area (25 mm^2^/200×) was measured with a “micrometer for microscope”.

### 2.6. Statistical Analysis

Data were presented as the mean ± SEM ($\bar{x}\pm s$). The independent sample t-test was used between the two groups, and differences between three or more groups were analyzed by one-way ANOVA and then multiple comparison tests (SPSS 21 software). A *p* < 0.05 was considered statistically significant.

## 3. Results

### 3.1. Establishment of Animal Models

As shown in Table 3, compared with the NC group, serum TC, TG, and LDL-C in the model group were significantly increased and HDL-C was significantly decreased (*p* < 0.05), indicating that the hyperlipidemia SD rat model was established successfully.

### 3.2. Effects of Different Intervention on Body Weight of Hyperlipidemia Rats

**Figure 1:**
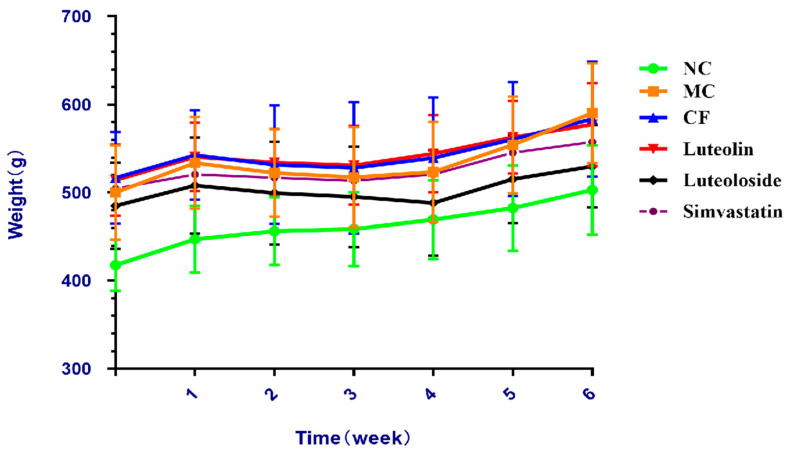
Body weight of different groups during intervention.

The body weight in each group increased gradually (Figure 1). From the 4th week, the weight growth rate of the model control group was significantly faster than that of the other four intervention groups.

### 3.3. Liver Index, Total Fat and Body Fat Ratio

The ratios of the liver and total fat to body weight were expressed as the relative weight per 100 g body weight (Table 4). The liver index and body fat ratio of the NC group were significantly lower than the MC group (*p* < 0.05). After 6 weeks, the CF, luteolin, and simvastatin groups were significantly lower than the MC group (*p* < 0.05). There were no significant differences between the three intervention groups and the simvastatin group.

### 3.4. Serum Lipid Concentrations of Each Groups

As shown in Table 5, the TG, TC, and LDL-C in serum in the MC group were significantly higher than that in the NC group, while the HDL-C was significantly lower (*p* < 0.05). After the intervention of CF, luteolin, luteoloside, and simvastatin, the level of TG, TC, and LDL-C in serum decreased significantly (*p* < 0.05), but there was no significant difference in HDL-C. Compared with the CF group, the simvastatin group was significantly lower (*p* < 0.05). There were no significant differences between the three intervention groups.

As shown in Table 6, the Apo-A1 in the MC group was significantly higher than that in the NC group. Apo-B in the MC group was higher than that in the NC group, but the difference was not statistically significant. Compared with the MC group, Apo-A1 in the CF group and luteoloside were significantly higher, and Apo-B in the CF group was significantly lower (*p* < 0.05). Compared with the simvastatin group, Apo-B in the CF group was significantly lower (*p* < 0.05). Compared with the CF group, Apo-B in luteoloside and the simvastatin group were significantly higher (*p* < 0.05).

### 3.5. Pathomorphological Features of Each Groups

**Figure 2:**
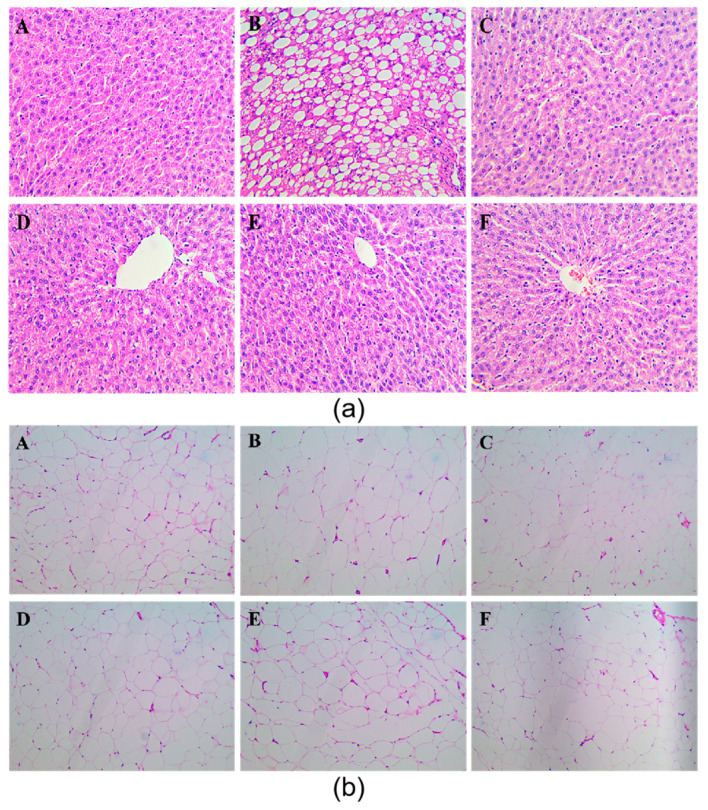
Pathological observation of liver (**a**) and peri-testicular fat (**b**) A:the normal control group(NC); (**B**): the model control group(MC); (**C**): Chrysanthemum flavone intervention group(CF); (**D**):Luteolin intervention group; (**F**): Luteoloside intervention group; (**E**): Simvastatin intervention group.

The pathomorphological features of liver and peri-testicular fat tissues were observed (Figure 2).

**Figure 3:**
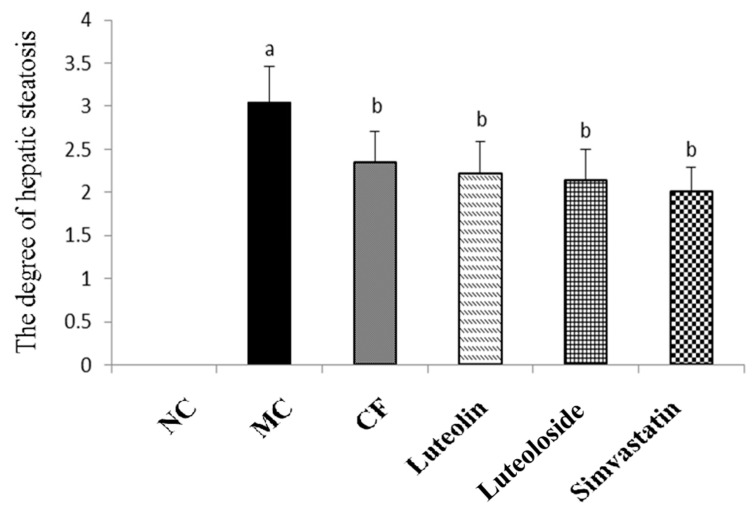
The steatosis score of liver in SD rats of each group. ^a^ Compared with NC, *p* < 0.05; ^b^ Compared with MC, *p* < 0.05.

Liver(a): The liver tissue of the NC group (A) was clear, and significant lipid droplets were not clearly observed. The hepatic sinuses of the MC group (B) disappeared and a large number of lipid droplets appeared. As shown in Figure 3, compared with the NC group, the degree of hepatic steatosis in the MC group was significantly increased (*p* < 0.05). After the intervention of chrysanthemum flavonoids (C), luteolin (D), Luteoloside (E), and simvastatin (F), the structure of hepatic sinuses were clear, and the lipid droplets were reduced. Compared with the MC group, the degree of lipidosis was significantly reduced (*p* < 0.05). The differences between the four intervention groups were not statistically significant.

Adipocytes(b): The adipocytes tissue of the NC group (A) had a clear outline and uniform size; compared with NC, adipocytes in the MC group (B) were uneven in size, with a general increase in the cross-sectional area and a tendency of fusion at some cell junctions. As shown in Table 7, compared with the NC group, the number of adipocytes in the MC group decreased significantly per 25 mm^2^ (*p* < 0.05). After the intervention of chrysanthemum flavonoids (C), luteolin (D), luteoloside (E), and simvastatin (F), the cross-sectional area of the adipocytes decreased, and the morphology of adipocytes became gradually uniform. Compared with the MC group, the number of adipocytes was significantly reduced (*p* < 0.05). There was no statistical difference in the number of adipocytes among the intervention groups.

### 3.6. Indicators of Liver Function in Serum

As shown in Table 8, compared with the NC group, the level of AST, ALT, and ALP in serum in the MC group increased significantly (*p* < 0.05). Compared with the MC group, AST, ALT, and ALP in the three intervention groups and the simvastatin group decreased significantly (*p* < 0.05), except AST in the luteolin group. There were no significant differences between the three intervention groups.

### 3.7. Antioxidant Levels in Livers

As shown in Table 9, compared with the NC group, the antioxidant level in liver tissue in the MC group decreased. In particular, CAT in liver tissue decreased and MDA increased (*p* < 0.05). Compared with the MC group, chrysanthemum flavonoids significantly improved the levels of GSH-Px and CAT (*p* < 0.05). Luteolin, luteoloside, and simvastatin all significantly decreased CAT and increased MDA (*p* < 0.05). Compared with the CF group, liver GSH-Px in the luteolin, luteoloside, and simvastatin groups were significantly decreased (*p* < 0.05), and there was no significant difference in other indicators among the three intervention groups.

### 3.8. Lipid Metabolism Related Enzyme Activities in Livers

As shown in Table 10, compared with the NC group, the liver levels of FAS, HMG-CoAR, and DGAT in the MC group increased, while FAβO, CYP7A1, and HL decreased significantly (*p* < 0.05). After the intervention of chrysanthemum flavonoids, luteolin, luteoloside, and simvastatin, the liver levels of FAS, HMG-CoA, and DGAT decreased significantly (*p* < 0.05), except for FAS in the luteoloside group. The liver levels of FAβO, CYP7A1, and HL increased significantly (*p* < 0.05), except for HL in the luteoloside group. Compared with the CF group, the differences of all liver enzymes in the luteoloside group were statistically significant (*p* < 0.05). Other measures did not differ statistically between the three intervention groups and the simvastatin group.

## 4. Discussion

As a metabolic disease with abnormal blood lipid levels, hyperlipidemia is an important risk factor for many complications and increases the incidence and mortality of cardiovascular diseases [23,24]. Although western medicine is the main method of treating hyperlipidemia, their limitations such as adverse reactions and intolerance are often reported, including musculoskeletal pain, elevation of transaminase, and headaches. Therefore, the lipid-lowering effect and application of traditional Chinese herbal medicine have gradually become the focus of many scholars [24]. The known phytochemicals with lipid-lowering effects mainly include phytosterols, phenols, saponins, alkaloids, organic sulfides, and lectins [25]. Chrysanthemum is a common edible herb in China and has been reported to contain antibacterial, antiviral, anti-oxidant, and immunomodulatory effects [26]. Some studies have shown that the ethyl acetate fraction of chrysanthemum indicum (CIEA) might be beneficial for preventing obesity [27,28,29], and in vitro chrysanthemum morifolium flower extract inhibits adipogenesis of 3T3-L1 cells via AMPK/SIRT1 pathway activation [30]. Luteolin and quercetin specifically inhibited NPC1L1 to reduce high blood cholesterol levels [31]. In addition, luteolin can also induce the expression of ABCG-5/8 in the intestinal mucosa of mice, also increasing fecal cholesterol content. Therefore, it is speculated that luteolin can treat hypercholesterolemia mainly by inhibiting cholesterol synthesis [32]. Luteolin release depends on glucosidase activity and affects the ability of an artichoke extract to inhibit the biosynthesis of cholesterol [33]. Glucoside was observed to improve blood lipids through glycogen synthase kinase 3 in a rat model of type 2 diabetes [34].

The results revealed that the body weight gains of rats in the model groups were significantly higher than the NC group, and the weight gains in the Chrysanthemum flavonoids, Luteolin, and Luteoloside groups were lower than that in the MC group (Figure 1). Organ coefficients were significantly improved in the three intervention groups and the simvastatin group, and there was no significant difference among these groups (Table 4). CF, Luteolin, Luteoloside, and simvastatin significantly reduced the body fat and lipid levels of hyperlipidemia rats, and simvastatin had a better effect on the reduction of low-density lipoprotein, while the other indicators showed no significant difference (Table 5). These results suggest that there was no significant difference in anti-obesity and blood lipids between them.

The emergence of the hypothesis of lipid accumulation and overflow explains the damage of hyperlipidemia to the body [35]. Hyperlipidemia disrupts the lipid balance and leads to increased TG synthesis and deposition in the liver, which is considered to be the first attack of the fatty liver [36]. In this study, the liver index in the NC groups was significantly higher than in the model group, which means that there may be some pathological changes, although there are certain limitations [37]. Chrysanthemum flavonoids, Luteolin, and Luteoloside all significantly improved the degree of hepatic steatosis in hyperlipidemia rats (Figure 2 and Figure 3). Studies have shown that the prolonged course of hyperlipidemia may be accompanied by different degrees of liver function damage, so the improvement of liver function also needs to be observed in the treatment of hyperlipidemia [38]. The three indexes of serum liver function in the model group were significantly increased, suggesting that the hyperlipidemia rats may be accompanied by a certain degree of liver damage (Table 7). Chrysanthemum flavonoids, Luteolin, Luteoloside, and simvastatin also had a protective effect on liver injury in the hyperlipidemia SD rats and there was no significant difference between them.

The “second strike theory” holds that the disorder of the antioxidant system further develops a fatty liver [39], which can be manifested as the increase in oxygen-free radical products and/or the decrease in the activity of free radical scavenging enzymes [40]. As antioxidant enzymes that remove reactive oxygen species in the body, SOD, GSH-Px, and CAT can reflect the antioxidant capacity of the body, together with the lipid peroxidation product MDA. The results showed that the antioxidant level in liver tissue decreased in hyperlipidemia SD rats. Chrysanthemum flavonoids, Luteolin, Luteoloside, and simvastatin can increase the activity of antioxidant enzymes and reduce lipid peroxidation products (Table 8). It is suggested that the effect of improving hepatic steatosis may be achieved by inhibiting lipid peroxidation and increasing the activity of antioxidant enzymes. However, the antioxidant activity of chrysanthemum flavonoids was better than that of the other three groups

The liver is an important player in regulating lipid metabolism. Imbalances of lipid metabolism in the liver can lead to non-physiological accumulation of triglycerides or steatosis [41,42]. Liver steatosis can be caused by complex processes: increased fatty acid uptake, synthesis of fat, and triglycerides combined with biogenesis or the growth of lipid droplets—LD catabolism decreased (including fatty acid oxidation) and the secretion of triglycerides or very-low-density lipoprotein (VLDL) was impaired [43]. Therefore, the activities of some lipid-metabolizing enzymes in the liver were detected from the aspects of synthesis and catabolism to explore the effects on liver lipid metabolism. Fatty acid synthase (FAS or FASN) plays a pivotal role in de novo lipogenesis, and functions as a central regulator of lipid metabolism [44,45]. Many FASN inhibitors have been successfully applied for the treatment of other diseases such as obesity, type 2 diabetes, and NAFLD [46]. FAβO is a key enzyme in fatty acid catabolism, which promotes the β-oxidation of fatty acids to regulate lipid metabolism [47]. Increased activation and decreased β-oxidation of fatty acids may lead to liver lipidosis [48]. Fatty acid metabolism was improved in hyperlipidemia rats fed with Chrysanthemum flavonoids, Luteolin, Luteoloside, and simvastatin. Chrysanthemum flavonoids may improve the activity of fatty acid metabolic enzymes better than others. Cholesterol 7α-hydroxylase (CYP7A1) is a rate-limiting enzyme that catalyzes the liver synthesis of bile acids, which converts cholesterol in non-hepatic peripheral tissues to bile acids [49]. The enzyme 3-hydroxy-3-methylglutaryl coenzyme A reductase (HMG-CoAR) is a rate-limiting enzyme of cholesterol synthesis, and its inhibitors are often used to treat hypercholesterolemia [50]. Chrysanthemum flavonoids, Luteolin, Luteoloside, and simvastatin can significantly improve the activity of cholesterol metabolism enzymes but Chrysanthemum flavonoids and luteolin may be better than the other two groups. Hepatic Lipase (HL) is an innate liver enzyme that promotes the clearance of TG from very-low-density lipoprotein (VLDL) pools, thereby regulating plasma TG content, but its release and transport are controlled by HDL [51]. Diacylglycerol acyltransferase (DGAT) is the final rate-limiting enzyme for the synthesis of triacylglycerol, mainly catalyzing the binding of diacylglycerol and fatty acids acyl. It is commonly used as a target for the treatment of obesity and diabetes [52]. The results showed that triglyceride metabolism was improved in hyperlipidemia rats fed with Chrysanthemum flavonoids, Luteolin, Luteoloside, and simvastatin. Chrysanthemum flavonoids and luteolin may be better than the other two groups.

## 5. Conclusions

In conclusion, Chrysanthemum flavonoids, Luteolin, and Luteoloside have anti-obesity effects and improve the serum lipids and fatty liver. The mechanism of action is related to the regulation of antioxidant levels and enzyme activities related to lipid metabolism in the liver. However, chrysanthemum flavonoids had a better effect on improving the antioxidant level and lipid metabolism enzyme activity. As hyperlipemia is a chronic disease, the intervention in this study is short, so the long-term intervention effect of Chrysanthemum flavonoids may be better. Further studies on other compounds related to this effect and the mechanism of action from the expression of genes and key proteins are needed.

## Figures and Tables

**Table 1:** Composition of the D12450B and D12492 diets (total calories were 4057 Kcal).

Component	10 % kcal-D12450B(g)	60 % kcal-D12492(g)
Casein, 30Mesh	200	200
cystine	3	3
Corn starch	315	0
maltodextrin	35	125
sucrose	350	68.8
Cellulose, BW200	50	50
Soybean oil	25	25
lard	20	245
The composite mineral s10026	10	10
dicalcium phosphate	13	13
calcium carbonate	5.5	5.5
potassium citrate, 1 H_2_O	16.5	16.5
Multi-VitaminsV10001	10	10
Choline tartrate	2	2
FD&C yellow #5	0.05	0.05

**Table 2:** Experimental groups and treatment doses.

Group	Dose of Gavage Administration	Diet
Normal control group	0.05 g/kg·bw/d 0.5% CMC solution	D12450B
Model control group	0.05 g/kg·bw/d 0.5% CMC solution	D12492
Chrysanthemum Flavonoids	100 mg/kg·bw/d Chrysanthemum Flavonoids [19]	D12492
Luteolin intervention group	50 mg/kg·bw/d Luteolin [20]	D12492
Luteoloside intervention group	25 mg/kg·bw/d Luteoloside [21]	D12492
Simvastatin intervention group	10 mg/kg·bw/d Simvastatin [22]	D12492

**Table 3:** Blood lipids in the normal control and model group rats after 4 weeks of modeling.

Group	TC(mmol/L)	TG(mmol/L)	HDL-C(mmol/L)	LDL-C(mmol/L)
Normal control group	2.05 ± 0.35	0.51 ± 0.14	0.42 ± 0.14	0.66 ± 0.17
Model group	2.42 ± 0.36 *	0.63 ± 0.16 *	0.33 ± 0.11 *	0.82 ± 0.21 *

* Compared with the normal control group, *p* < 0.05.

**Table 4:** Body weight, liver weight, liver index, total fat, and body fat ratio in each group.

Group	Liver Weight (g)	Total Fat (g)	Body Weight (g)	Liver Index (%)	Body Fat Ratio (%)
NC	10.87 ± 1.47	23.51 ± 7.95	503.00 ± 50.47	2.13 ± 0.21	4.88 ± 1.54
MC	14.48 ± 1.21 ^a^	43.06 ± 11.64 ^a^	596.67 ± 56.26 ^a^	2.64 ± 0.16 ^a^	7.40 ± 1.28 ^a^
CF	12.57 ± 1.27 ^b^	33.53 ± 10.70 ^b^	583.50 ± 65.43 ^a^	2.15 ± 0.26 ^b^	5.82 ± 0. 83 ^b^
Luteolin	12.20 ± 1.15 ^b^	32.32 ± 8.97 ^b^	577.30 ± 46.86 ^a^	2.12 ± 0.18 ^b^	5.62 ± 1.41 ^b^
Luteoloside	11.51 ± 0.94 ^b^	28.40 ± 6.95 ^b^	529.80 ± 46.76 ^b^	2.17 ± 0.06 ^b^	5.36 ± 1.24 ^b^
Simvastatin	11.36 ± 0.75 ^b^	29.10 ± 6.59 ^b^	540.83 ± 30.63 ^b^	2.10 ± 0.15 ^b^	5.11 ± 0.98 ^b^

^a^ Compared with NC, *p* > 0.05; ^b^ Compared with MC, *p* < 0.05.

**Table 5:** The serum levels of TG, TC, HDL-C, and LDL-C in serum in each group.

Group	TC(mmol/L)	TG(mmol/L)	LDL-C(mmol/L)	HDL-C(mmol/L)
NC	1.95 ± 0.33	0.68 ± 0.12	0.42 ± 0.09	1.02 ± 0.29
MC	2.74 ± 0.63 ^a^	1.42 ± 0.53 ^a^	0.68 ± 0.15 ^a^	0.82 ± 0.12 ^a^
CF	2.23 ± 0.32 ^b^	0.69 ± 0.21 ^b^	0.50 ± 0.08 ^b^	0.84 ± 0.19
Luteolin	2.05 ± 0.24 ^b^	0.52 ± 0.13 ^b^	0.45 ± 0.04 ^b^	0.87 ± 0.15
Luteoloside	2.05 ± 0.39 ^b^	0.48 ± 0.06 ^b^	0.43 ± 0.12 ^b^	0.85 ± 0.20
Simvastatin	2.04 ± 0.28 ^b^	0.57 ± 0.10 ^b^	0.39 ± 0.04 ^b, c^	0.87 ± 0.11

^a^ Compared with NC, *p* < 0.05; ^b^ Compared with MC, *p* < 0.05; ^c^ Compared with CF, *p* < 0.05.

**Table 6:** Changes of Apo-A1, Apo-B, and Apo-B/Apo-A1 in SD rats of each group.

Group	Apo-A1(ng/mL)	Apo-B(ng/mL)	Apo-B/Apo-A1
NC	20.20 ± 2.80	59.64 ± 3.04	3.03 ± 0.35
MC	15.52 ± 3.01 ^a^	64.00 ± 9.28	4.00 ± 1.23
CF	20.32 ± 3.72 ^b^	50.32 ± 9.55 ^b^	2.93 ± 0.91
Luteolin	18.17 ± 0.95	60.37 ± 8.23	2.50 ± 0.28
Luteoloside	20.54 ± 6.93 ^b^	62.87 ± 4.86 ^c^	3.50 ± 1.63
Simvastatin	18.88 ± 3.92	61.73 ± 4.96 ^c^	3.44 ± 0.68

^a^ Compared with NC, *p* < 0.05; ^b^ Compared with MC, *p* < 0.05; ^c^ Compared with CF, *p* < 0.05.

**Table 7:** Changes in the number of adipocytes in each group.

Group	Number of Adipocytes (per 25 mm^2^, 200×)
NC	16.8 ± 1.3
MC	12.5 ± 1.7 ^a^
CF	16.2 ± 2.1 ^b^
Luteolin	15.9 ± 2.5 ^b^
Luteoloside	15.1 ± 2.4 ^b^
Simvastatin	15.6 ± 1.9 ^b^

^a^ Compared with NC, *p* < 0.05; ^b^ Compared with MC, *p* < 0.05.

**Table 8:** Changes of AST, ALT, and ALP in the serum of each group.

Group	AST(IU/L)	ALP(IU/L)	ALT(IU/L)
NC	135.29 ± 16.94	106.11 ± 17.81	39.78 ± 8.76
MC	161.50 ± 33.20 ^a^	203.40 ± 66.89 ^a^	57.10 ± 14.10 ^a^
CF	125.50 ± 28.75 ^b^	132.60 ± 48.87 ^b^	49.87 ± 10.06 ^b^
Luteolin	142.22 ± 19.72	118.40 ± 15.35 ^b^	45.30 ± 10.43 ^b^
Luteoloside	126.25 ± 15.28 ^b^	105.40 ± 19.55 ^b^	39.60 ± 7.44 ^b^
Simvastatin	126.00 ± 22.42 ^b^	120.00 ± 33.88 ^b^	41.57 ± 5.44 ^b^

^a^ Compared with NC, *p* < 0.05; ^b^ Compared with MC, *p* < 0.05.

**Table 9:** GSH-Px, CAT, SOD, and MDA in the livers of each group.

Group	GSH-Px(U/mgprot)	CAT(U/mgprot)	SOD(U/mgprot)	MDA(nmol/mL)
NC	913.98 ± 275.71	51.81 ± 14.33	199.70 ± 35.54	1.07 ± 0.18
MC	665.14 ± 313.03	33.31 ± 5.08 ^a^	186.56 ± 42.59	1.48 ± 0.38 ^a^
CF	1553.13 ± 306.73 ^a, b^	52.65 ± 11.85 ^b^	229.00 ± 47.41	1.26 ± 0.32
Luteolin	980.99 ± 380.80 ^c^	51.46 ± 14.12 ^b^	202.26 ± 57.03	1.1 ± 0.34 ^b^
Luteoloside	975.52 ± 212.04 ^c^	63.11 ± 16.26 ^b^	204.36 ± 46.71	1.07 ± 0.13 ^b^
Simvastatin	891.71 ± 348.21 ^c^	60.95 ± 16.84 ^b^	184.01 ± 37.53	0.9 ± 0.18 ^b^

^a^ Compared with NC, *p* < 0.05; ^b^ Compared with MC, *p* < 0.05; ^c^ Compared with CF, *p* < 0.05.

**Table 10:** Enzymes involved in fatty acid (FAS, FAβO), cholesterol (HMG-CoAR, CYP7A1), and triglycerides (DGAT, HL) metabolism in the liver.

Group	FAS (nmol/g)	FaβO (pg/g)	HMG-CoAR(ng/g)	CYP7A1 (ng/g)	DGAT(ng/g)	HL(μmol/g)
NC	0.039 ± 0.004	12261.07 ± 992.49	55.11 ± 2.83	34.96 ± 2.71	15.55 ± 1.89	1.30 ± 0.08
MC	0.055 ± 0.005 ^a^	8198.48 ± 799.24 ^a^	76.43 ± 4.25 ^a^	23.40 ± 1.59 ^a^	25.26 ± 1.90 ^a^	1.03 ± 0.07 ^a^
CF	0.044 ± 0.004 ^a, b^	11892.12 ± 695.43 ^b^	61.11 ± 4.29 ^a, b^	32.18 ± 1.47 ^a, b^	17.61 ± 2.11 ^a, b^	1.21 ± 0.06 ^a, b^
Luteolin	0.048 ± 0.005 ^a, b^	10500.34 ± 904.70 ^a, b, c^	63.69 ± 3.90 ^a, b^	30.57 ± 2.66 ^a, b^	18.32 ± 1.66 ^a, b^	1.23 ± 0.07 ^a, b^
Luteoloside	0.050 ± 0.004 ^a, c^	9489.81 ± 1118.32 ^a, b, c^	69.94 ± 2.99 ^a, b, c^	26.55 ± 2.87 ^a, b, c^	21.47 ± 0.64 ^a, b, c^	1.03 ± 0.09 ^a, c^
Simvastatin	0.049 ± 0.004 ^a, b^	9851.16 ± 873.80 ^a, b, c^	71.02 ± 5.25 ^a, b, c^	26.02 ± 1.94 ^a, b, c^	19.67 ± 1.70 ^a, b, c^	1.19 ± 0.07 ^a, b^

^a^ Compared with NC, *p* < 0.05; ^b^ Compared with MC, *p* < 0.05; ^c^ Compared with CF, *p* < 0.05.

## Data Availability

The data presented in this study are available on request from the corresponding author. The data are not publicly available due to it involves some of the genetic data from our later mechanistic studies.
